# miR-125 inhibits colorectal cancer proliferation and invasion by targeting TAZ

**DOI:** 10.1042/BSR20190193

**Published:** 2019-12-13

**Authors:** Meiyuan Yang, Xiaoli Tang, Zheng Wang, Xiaoqing Wu, Dong Tang, Daorong Wang

**Affiliations:** 1 Department of General Surgery, The Second Xiangya Hospital of Central South University, Renmin Road No.139, Changsha 410001, China; 2 Department of General Surgery, Clinical Medical College of Yangzhou University, Huaihai Road No.7, Yangzhou 225001, China; 3 Department of General Surgery, Medical College, Yangzhou University, Northern Jiangsu Province Hospital, General Surgery Institute of Yangzhou University, Nantong Road No.98, Yangzhou 225001, China

**Keywords:** colorectal neoplasm, invasion, miR-125, proliferation, TAZ

## Abstract

Colorectal cancer (CRC) is the third most common malignant tumor worldwide and is a serious threat to human health. MicroRNAs (miRNAs) play a key role in oncogenesis and cancer progression. MiRNA-125 (miR-125) is an important miRNA that is dysregulated in several kinds of cancers. Thus, we investigated the expression and effects of miR-125 and Transcriptional co-activator with PDZ-binding motif (TAZ) for a better understanding of the underlying mechanism of tumor progression in CRC, which may provide an emerging biomarker for diagnosis and treatment of CRC. We measured the expression levels of miR-125 in CRC tissues, adjacent tissues, and cell lines (e.g. HCT116, SW480, FHC) by quantitative real-time polymerase chain reaction (qRT-PCR). The effect of miR-125 on proliferation and invasion in CRC cells was detected by Cell Counting Kit-8 (CCK-8), clone formation assay, and transwell assay. Western blotting and qRT-PCR were used to investigate the expression of TAZ after knocking down miR-125 in HCT116 cells or overexpressing miR-125 in SW480 cells. MiR-125 was significantly down-regulated in CRC compared with pericarcinomatous tissue from 18 patients. An miR-125 inhibitor promoted CRC cell proliferation and invasion, while miR-125 mimic had the opposite effect. Moreover, we found that TAZ was an miR-125 target and the siRNA knockdown of TAZ could reverse the effect of the miR-125 inhibitor on proliferation and invasion in HCT116 cells. The present study shows that miR-125 suppresses CRC proliferation and invasion by targeting TAZ.

## Introduction

Colorectal cancer (CRC) is the third most commonly diagnosed cancer in males and females, with an estimated 145600 new cases and 51020 deaths occurring worldwide in 2019 [1]. Although a study has shown that CRC incidence and mortality rates declined over the past few decades due to the popularization of colonoscopy, advanced CRC still severely threatens human health [2]. Therefore, it is vital to study the underlying molecular mechanisms which may help find a more effective biomarker to detect tumorigenesis.

MicroRNAs (miRNAs) are small, non-protein coding RNAs that suppress gene expression by binding complementary sequences in the 3′-untranslated regions (3′-UTR) and then affecting mRNA stability or interfering with protein translation [3]. Croce and Calin [4] concluded that miRNA may contribute to tumorigenesis after reviewing several studies. Several reports indicate that miRNAs affect tumorigenesis and progression, including liver cancer, cervical cancer, and CRC [5–7]. MicroRNA-125 (miR-125) is a tumor suppressor, which was down-regulated in some tumors, such as bladder cancer, breast cancer, lung cancer, and hepatocellular carcinoma [8–12]. MiR-125 has also been found to be down-regulated in CRC tissues compared with adjacent normal tissues and may play a suppressive role in the development of CRC [13–15].

Hippo signaling modulates cell proliferation and apoptosis through downstream targets or by interacting with other pathways, such as Wnt and MAPK signaling [16,17]. Yes-associated protein (YAP) and transcriptional co-activator with PDZ-binding motif (TAZ) are two main effectors in the Hippo signaling pathway which regulate organ development, cell self-renewal, and tumorigenesis [18]. Diamantopoulou et al. [19], reported that a decrease in TAZ and YAP could suppress CRC invasion and migration.

We identified TAZ is a potential target of miR-125 through predictions based on TargetScan, miRTarBase, and miRDB. We then further investigated the role of miR-125 and TAZ in CRC and its effect on proliferation and invasion.

## Materials and methods

### Patient samples and cell cultures

Frozen CRC tissues and matched pericarcinomatous tissues were obtained from 18 patients who underwent a CRC resection at Northern Jiangsu People’s Hospital during 2016–2017. Before collecting and using the specimens, all patients provided informed consent and the experiment conformed to the World Medical Association Declaration of Helsinki. Additionally, ethical approval was obtained from the Medical Ethics Committee of Northern Jiangsu People’s Hospital. The specimens were immediately immersed in liquid nitrogen once obtained from patients. The human CRC cell lines HCT116, SW480, and HEK293 were purchased from the Shanghai Institute for Biological Sciences, Chinese Academy of Science. The human colon epithelial cell line (FHC) was purchased from American Type Culture Collection. HCT116 and HEK293 cells were cultured in DMEM (Corning, U.S.A.) with 1% glutamine. While SW480 was cultivated in L15 (Corning, U.S.A.) and FHC in DMEM/F12 (Corning, U.S.A.). All culture media contained 10% fetal bovine serum (Ever Green, China) and 1% penicillin/streptomycin (Invitrogen, U.S.A.). The cells were incubated in 5% CO_2_ and the temperature maintained at 37°C

### RNA extraction, inverse transcription, and quantitative real-time polymerase chain reaction

Total RNA was extracted from tissue samples and cell lines (HCT116, SW480, FHC) by using MiPure Cell/Tissue miRNA Kit (Vazyme, China). The process to synthesize cDNA followed the manufacturer’s specification of miRNA First Strand cDNA Synthesis Kit (Vazyme, China). Then, a quantitative reverse-transcription polymerase chain reaction (qRT-PCR) was performed with the cDNA as a template using miRNA universal SYBR qPCR Master Mix (Vazyme, China). The expression levels of miR-125 and TAZ mRNA were calculated by using the 2^−ΔΔ*C*_T_^ method, which were normalized to U6 expression. Primers were also needed in the amplification process. The sequence from 5′–3′ is as follows. U6: Forward, CTCGCTTCGGCAGCACA; Reverse, AACGCTTCACGAATTTGCGT; miR-125: Forward, GGGTCCGAGGTATTCGCACT; Reverse, TCCCTGAGACCCTTTAACCTGTG; TAZ: Forward, CACCGTGTCCAATCACCAGTC; Reverse, TCCAACGCATCAACTTCAGGT.

### *In silico* analysis

The binding site of miR-125 and TAZ was analyzed by TargetScan (http://www.targetscan.org/vert_72/), miRDB (http://www.mirdb.org/), miRTarBase (http://mirtarbase.mbc.nctu.edu.tw/php/index.php). We collected target gene from public database for further study.

### Transfections

The miR-125 mimic, miR-125 inhibitor, and TAZ-siRNA were purchased from RiboBio (China) to regulate the expression of miR-125 and TAZ. Based on the manufacturer’s instructions, FECT™ CP (RiboBio, China) was used to transfect miR-125 mimic, miR-125 inhibitor, and siRNA into SW480 and HCT116 cells. Finally, cells were collected to detect gene and protein expression 48 h after transfection.

### Cell counting kit-8 assay

Cell proliferation was detected by cell counting kit-8 (CCK-8) assay (Dojindo, Japan). First, 2 × 10^3^ HCT116 cells were seeded into 96-well plates and incubated for 24 h at 37°C. Then, cells were mixed with 10 μl cck-8 reagent and subsequently incubated for 90 min on five consecutive days. An automatic microplate reader (BioTek, U.S.A.) was used to measure OD and, incubation and measurement of OD value was performed at the same time everyday.

### Clone formation assay

The clone formation assay involved seeding 4 × 10^2^ HCT116 and SW480 cells in six-well plates which were cultured in 2 ml complete media. After cultivating for 12 days, the cells were fixed in 4% fixative solution (Solarbio, China) for 15 min and dyed using 1 ml Crystal Violet for 20 min and 2 ml PBS was used to wash out excess dye. We then calculated the number of clones formed.

### Transwell assay

Transwell chambers (Corning) with 8.0 μm pores in the bottom membrane were placed in 24-well plates. After adding 300 µl serum-free medium to hydrate the matrigel for 30 min, the upper chamber was seeded with 1 × 10^5^ cells in serum-free media, while 500 µl complete medium was added to the corresponding lower chamber, fetal calf serum as a driving force to attract cell movement. One milliliter Crystal Violet solution was used to stain the cells after cultivating them for 48 h. Then, the cells were washed twice with PBS and upper bottom cells were wiped off with cotton swabs. We counted the migratory cells in the upper chamber.

### Western blot

Proteins were obtained using a protein extraction kit (Beyotime, China) according to manufacturers’ instructions. Then, proteins were resolved by 10% SDS/PAGE gel and transferred to 0.22 µm polyvinylidene difluoride (PVDF) membranes (Millipore, U.S.A.). Membranes were incubated with blocking buffer (5% skim milk + 100 ml TBST) at 37°C. Afterward, membranes were incubated with anti-TAZ antibody (Proteintech, China) overnight at 4°C, and incubated with HRP-linked secondary antibodies (Bioworld, U.S.A.) for 1 h at 37°C. The chemiluminescence method was used to detect the antibodies in the experiment (Bio-Rad, U.S.A.). Glyceraldehyde 3-phosphate dehydrogenase (GAPDH) was used as an internal reference.

### Dual-luciferase reporter assay

The wild-type (wt) and mutant (mut) reporter plasmids of TAZ 3′UTR were constructed by RiboBio (Guangzhou, China) and then cloned into the pmiR-RB-REPORT vector. The miR-125 mimic and vector were co-transfected into HEK293 cells and the NC group using the same method. After incubation for 48 h, cells were collected. Luciferase activity was tested by promega (Madison, U.S.A.) according to the manufacturers’ instructions. *Renilla* luciferase activity was regarded as an internal control.

### RNA immunoprecipitation assay

Magna RIP RNA Binding Protein Immunoprecipitation Kit (Millipore, U.S.A.) was used to perform RNA immunoprecipitation (RIP) assay. First, the complete RIP lysis buffer was formulated with 100 µl RIP lysis buffer, 0.5 µl protease inhibitor mixture and 0.25 µl Rnase inhibitor, and then cells were lysed using the complete RIP lysis buffer for 1 h. Next, the RNA bound protein complex was precipitated using A/G magnetic beads. Then protease K was applied to the samples after using magnetic frame fixation. At last, the RNA obtained was used for qRT-PCR analysis.

### Statistical analysis

GraphPad Prism 7.0 was used to analyze the data, and all the experiments were repeated at least three times and mean ± standard deviation (SD) was calculated to hand duplicate data. Appropriate statistical methods were applied to compare the differences between groups and they were based on statistical principles. *P*<0.05 is considered statistically significant.

## Results

### The expression of miR-125 is decreased in CRC

**Figure 1:**
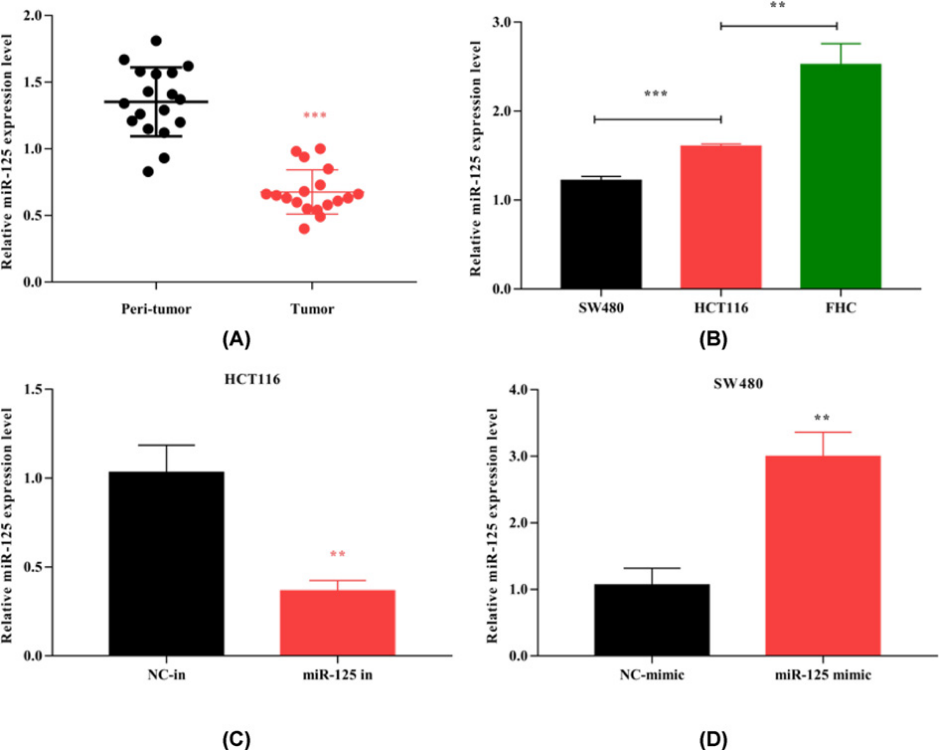
The expression of miR-125 is decreased in CRC tissues and cell lines (**A**) The expression level of miR-125 in CRC tissues and corresponding normal tissues by qRT-PCR (*n*=18). (**B**) Relative expression of miR-125 in CRC cell lines by qRT-PCR (SW480, HCT116 and FHC). (**C,D**) miR-125 inhibitor transiently transfects HCT116, while miR-125 mimic transfects SW480, relative miR-125 level is detected in HCT116 and SW480 when transfected after 48 h (mean ± SD is applied to process three repeat data; **P<0.01, ***P<0.001).

CRC tissues and adjacent normal tissues from 18 patients were used to detect the expression of miR-125. We found that miR-125 was down-regulated in CRC by qRT-PCR analysis (Figure 1A). Reduction in miR-125 expression has been reported in CRC [13–15]. Then, we studied the expression of miR-125 in two common CRC cell lines (SW480 and HCT116) and a normal human colon epithelial cell line (FHC), which showed that the levels of miR-125 in FHC cells were higher than in SW480 and HCT116 cells, and expression in SW480 was lower than HCT116 cells (Figure 1B). The miR-125 inhibitor was transfected into HCT116 cells and the miR-125 mimic into SW480 cells. Figure 1C,D show that miR-125 was significantly knocked down in HCT116 cells and overexpressed in SW480 cells.

### The expression of miR-125 inhibits CRC cell lines proliferation and invasion

**Figure 2:**
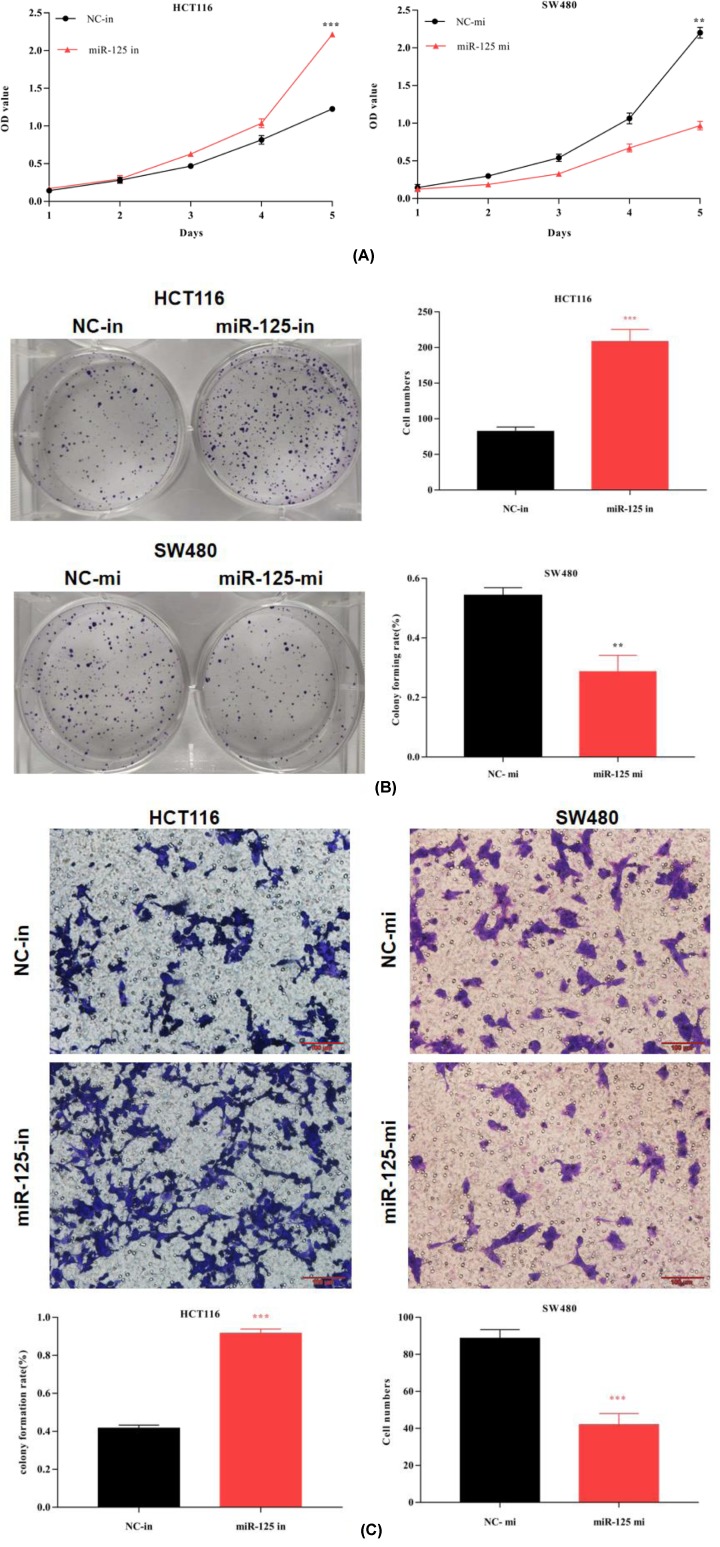
Aberrant expression of miR-125 controls HCT116 and SW480 proliferation and metastases (**A**) CCK-8 assay indicates that the OD value of miR-125 inhibitor is higher than NC. While miR-125 mimic is the opposite. (**B**) Clone formation rates increase by miR-125 inhibitor in HCT116, while miR-125 mimic decrease that. (**C**) Transwell assay for cell metastases shows that miR-125 inhibitor accelerate the process of metastases, while miR-125 mimic slows that (all experiments are repeated three times; **P<0.01, ***P<0.001).

To explore the role of miR-125 in CRC, we validated the inhibitory effect of miR-125 on proliferation and invasion in cell lines. CCK-8 assay was used to analyze cell proliferation. In agreement, we found that the OD value of HCT116 cells transfected with the miR-125 inhibitor were higher than the control group, while the miR-125 mimic prevented this increase (Figure 2A).

Clone formation assay was another method used to observe the proliferative capacity. The results were consistent with the previous CCK-8 assay results. Overexpression of miR-125 significantly decreased clone formation in SW480 cells, whereas the opposite results were observed in HCT116 cells with knockdown of miR-125 (Figure 2B).

Next, we employed a transwell assay to evaluate cell invasion. Transwells with matrigel is a classic assay to assess cell invasion. As showed in Figure 2C, HCT116 cells transfected with miR-125 inhibitor had higher invasion rates in contrast with the negative control. Meanwhile, the miR-125 mimic cells reduced invasive ability. Thus, these results suggested that miR-125 inhibited CRC cell proliferation and invasion.

### TAZ is a downstream target of miR-125

**Figure 3:**
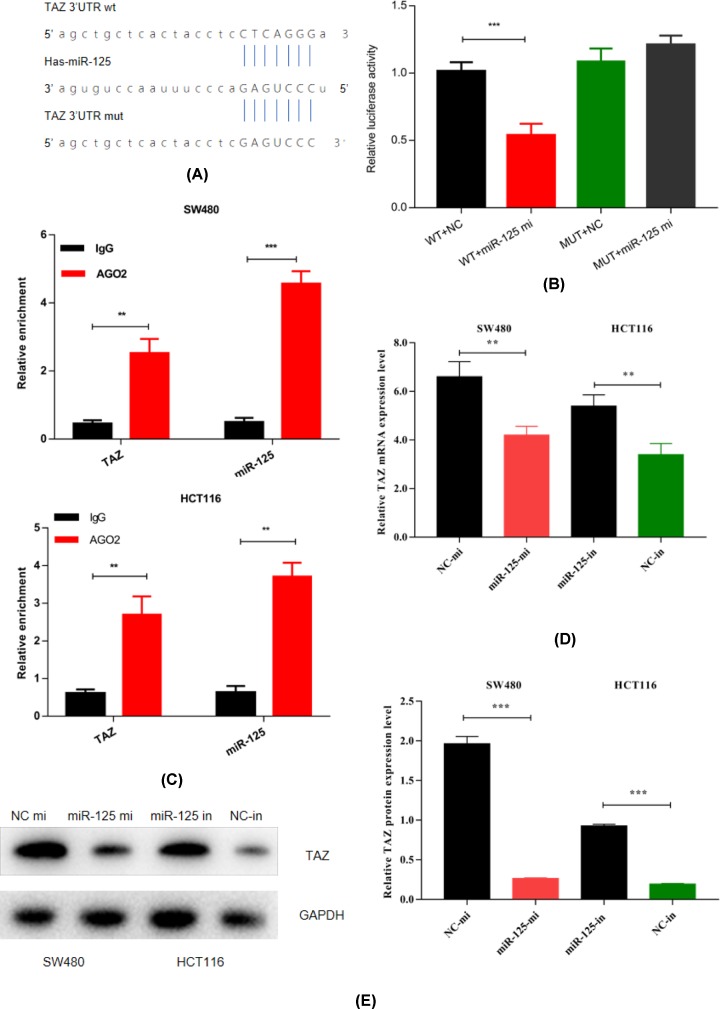
TAZ is a target of miR-125 in HCT116 and SW480 cells (**A**) The binding site of miR-125 and TAZ 3′-UTR *in silico* analysis. (**B**) The luciferase activity of plasmid that transfected pGL3-TAZ-wt was significantly reduced, while the other group did not find that. (**C**) RIP assay confirmed that miR-125 and TAZ were conjunct in HCT116 and SW480 cell lines. (**D**) qRT-PCR analysis of TAZ mRNA level in miR-125 mimic, miR-125 inhibitor and control group. (**E**) TAZ protein level in miR-125 mimic, miR-125 inhibitor and NC by Western blot, GAPDH is an internal reference (all data are representative of three experiments in triplicate; **P<0.01, ***P<0.001).

TAZ has been reported to be a potential target of miR-125 [20–22]. We utilized bioinformatics analysis (TargetScan, miRDB, miRTarBase) to predict the potential targets. The binding site of miR-125 and TAZ is shown in Figure 3A. Although the regulation of TAZ by miR-125 has been reported, there was a lack of evidence to prove the relationship between miR-125 and TAZ in CRC. Therefore, the mRNA and protein level of TAZ was measured by qRT-PCR and Western blotting, and we found that miR-125 could negatively regulate the expression of TAZ. As seen in Figure 3D,E, TAZ mRNA and protein levels were reduced in the miR-125 mimic transfected cells in comparison with the negative control, while the miR-125 inhibitor had the opposite effect. To further verify the relationship between miR-125 and TAZ, dual-luciferase and RIP assay was performed. The luciferase activity was significantly reduced in cells co-transfected with miR-125 mimic and wild-type. The phenomenon was not observed in the NC group which was transfected with pGL3-TAZ-mut (Figure 3B). Additionally, RIP assay was performed to confirm this relationship, as shown in Figure 3C. We found that miR-125 and TAZ were significantly enriched in Ago2-binding beads compared with the input group by qRT-PCR analysis.

### TAZ down-regulation could reverse miR-125 inhibitory role in CRC

**Figure 4:**
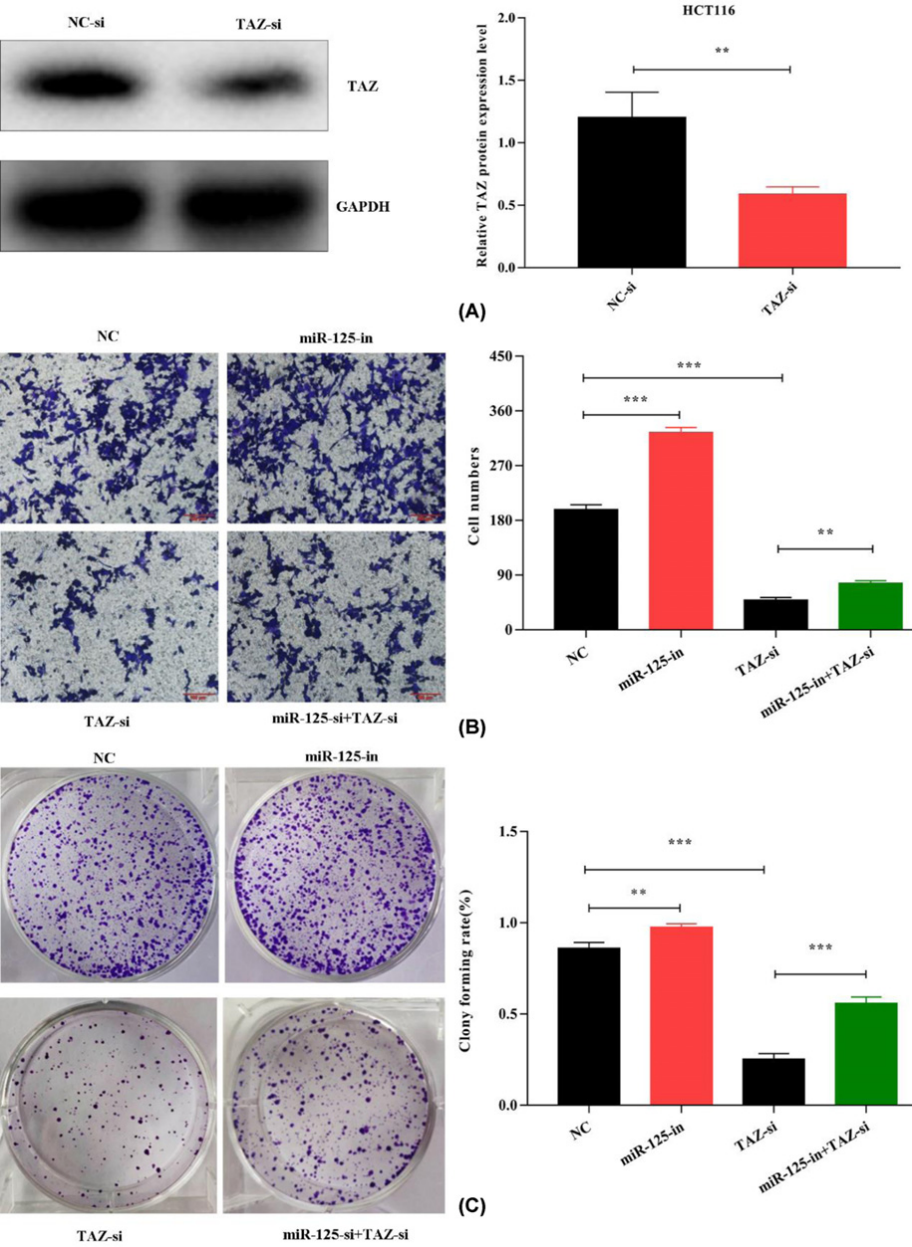
TAZ down-regulation could reverse miR-125 inhibitory role in CRC (**A**) TAZ-siRNA is transfected to HCT116. The expression of TAZ protein is decreased in siRNA of TAZ by Western blot. (**B**) Down-regulated TAZ reverses the effect of miR-125 inhibitor on metastases on HCT116 cell. (**C**) Clone formation assay showed that miR-125 inhibitory role was restored by TAZ-siRNA (all experiments are repeated three times; **P<0.01, ***P<0.001).

To further confirm that miR-125 regulates proliferation and invasion by targeting TAZ, we transfected TAZ-siRNA into HCT116 cells. Western blotting was used to confirm the lower expression of TAZ (Figure 4A). The miR-125 inhibitor and TAZ-siRNA were co-transfected into HCT116. Using the clone formation and transwell assays, we observed that TAZ-siRNA could reverse the effect of miR-125 inhibitor on proliferation and invasion (Figure 4B,C). Thus, we think that miR-125 controls CRC proliferation and invasion by targeting TAZ.

## Discussion

In our study, we analyzed the expression of miR-125 in primary CRC tissues and related cancer cell lines. Experimental data support that miR-125 plays a tumor suppressor role in CRC. We confirmed TAZ as a target of miR-125, which was predicted by *in silico* analysis and verified by qRT-PCR, Western blotting, dual-luciferase reporter, and RIP assay. MiR-125 suppresses CRC cell proliferation and invasion by targeting TAZ.

MiRNAs, as pivotal regulators of gene expression, affect tumorigenesis and cancer progression [23]. Studies have shown that miR-125 was differentially expressed in various cancers. MiR-125 was down-regulated in gastric cancer and hepatocellular carcinoma, while it was up-regulated in esophageal cancer and head and neck squamous cell carcinoma [24–27]. The mechanisms by which miR-125 regulate CRC forms a complex network, miR-125 could negatively modulate BCL2, BCL2L12, and Mcl-1, and down-regulated miR-125 promoted colon cancer cell proliferation and inhibited apoptosis [15]. Dual-luciferase reporter assay was used to validate vascular endothelial growth factor (VEGF) and vascular endothelial growth factor A (VEGFA) binding of miR-125 and could reverse the inhibitory influence caused by miR-125 overexpression [13,28]. Regulating signaling pathways is another way that miR-125 regulates CRC progression whereby miR-125 controlled CRC development by targeting fucosyltransferase 5/fucosyltransferase 6 (FUT5/FUT6) and modulating the PI3K-AKT signaling pathway [29]. MiR-125 not only affects the phenotype of CRC by regulating downstream targets but is also modulated by upstream regulatory factors. LncRNA may act as an miR-125 sponge and the HOXA11-AS-miR-125-PADI2 regulatory network is involved in liver metastases of CRC (CRLM) [30]. Thus, miR-125 acted as a tumor inhibitor in CRC according to our study.

Hippo signaling pathway modulates TAZ and YAP by limiting their entry into the nucleus and the pathway plays a crucial role in cancer cell invasion, proliferation, and metastases [31]. According to previous studies, TAZ was a target of different miRNAs in various cancers, including lung cancer, liver cancer, and gastric cancer [32–34]. In mice experiments, TAZ accelerated acinar-to-ductal metaplasia and which lead to pancreatic cancer by activating the JAK-STAT3 signaling pathway [35]. TAZ promoted tumorigenesis and progression, further affecting prognosis.

The relationship between miR-125 and TAZ has been reported in other cancers and was identified by qRT-PCR, Western blotting, and dual-luciferase reporter gene assay. In these tumors, miR-125 was reduced in tumor tissues compared with normal tissues and had a negative regulatory correlation with TAZ [15,20,21].

To our knowledge, this is the first report trying to explain the relationship between miR-125 and TAZ in CRC but there are certain limitations in our study. First, we only select one target (TAZ) to validate the effect of miR-125. MiR-125 may regulate proliferation and invasion by acting on various targets rather than solely on TAZ. Second, although we have tested the expression of miR-125, the study lacks a larger clinical sample validation. Therefore, we failed to verify the relationship between miR-125, clinical prognosis, and pathological characteristics.

Although our study has some deficiencies, it could provide some insight into clinical applications. Here we have shown that the expression of miR-125 was decreased in CRC and modulated proliferation and invasion by targeting TAZ, suggesting miR-125 may be an emerging biomarker for CRC patients. Prior studies have reported that the expression of circulating miR-125a was decreased in Crohn’s disease and circulating exosomal miR-125a-3p was deemed to have the potential to screen for early-stage CRC [36,37]. Moreover, previous research has shown that TAZ has a prognostic value in evaluating relapse and survival in patients with CRC. The expression of TAZ increased in CRC, promoting cell proliferation and epithelial–mesenchymal transition [38,39]. MiR-125 and its target TAZ could be a prognostic marker for CRC. We hope to further explore the miR-125-regulated signaling pathways and the upstream regulatory factors of miR-125 in CRC.

In conclusion, miR-125 inhibits CRC proliferation and invasion by targeting TAZ. MiR-125 and TAZ might be emerging biomarkers for diagnosis and prognosis in CRC.

## Abbreviations

AGO2: argonaute 2
BCL2: B cell lymphoma-2
BCL2L12: B cell lymphoma-2 like protein-12
CCK-8: cell counting kit-8
CRC: colorectal cancer
HRP: hrp horseradish peroxidase
MCL1: myeloid cell leukemia-1
miRNA: microRNA
miR-125: microRNA-125
NC: negative control
OD: optical density
qRT-PCR: quantitative real-time polymerase chain reaction
RIP: RNA immunoprecipitation
TAZ: transcriptional co-activator with PDZ-binding motif
TBST: tris-buffered saline tween
YAP: yes-associated protein
